# Interplay between YAP/TAZ and metabolic dysfunction-associated steatotic liver disease progression

**DOI:** 10.1007/s12272-024-01501-5

**Published:** 2024-06-14

**Authors:** Na Young Lee, Myeung Gi Choi, Eui Jin Lee, Ja Hyun Koo

**Affiliations:** 1 College of Pharmacy, Seoul National University, Seoul, 08826 Korea; 2 Research Institute of Pharmaceutical Sciences and Natural Products Research Institute, Seoul National University, Seoul, 08826 Korea

**Keywords:** Liver disease, MASLD, YAP/TAZ, Hippo pathway

## Abstract

Metabolic dysfunction-associated steatotic liver disease (MASLD) is becoming an increasingly pressing global health challenge, with increasing mortality rates showing an upward trend. Two million deaths occur annually from cirrhosis and liver cancer together each year. Yes-associated protein (YAP) and transcriptional coactivator with PDZ-binding motif (TAZ), key effectors of the Hippo signaling pathway, critically regulate tissue homeostasis and disease progression in the liver. While initial studies have shown that YAP expression is normally restricted to cholangiocytes in healthy livers, the activation of YAP/TAZ is observed in other hepatic cells during chronic liver disease. The disease-driven dysregulation of YAP/TAZ appears to be a critical element in the MASLD progression, contributing to hepatocyte dysfunction, inflammation, and fibrosis. In this study, we focused on the complex roles of YAP/TAZ in MASLD and explored how the YAP/TAZ dysregulation of YAP/TAZ drives steatosis, inflammation, fibrosis, and cirrhosis. Finally, the cell-type-specific functions of YAP/TAZ in different types of hepatic cells, such as hepatocytes, hepatic stellate cells, hepatic macrophages, and biliary epithelial cells are discussed, highlighting the multifaceted impact of YAP/TAZ on liver physiology and pathology.

## Introduction

Yes-associated protein (YAP) and transcriptional coactivator with PDZ-binding motif (TAZ) are key effectors of the Hippo signaling pathway and play vital roles in regulating cell fate decisions, tissue growth, regeneration, and metabolism cell proliferation (Maglic et al. 2018; Koo et al. 2020; Daoud et al. 2021). With a strong evolutionary conservation, their roles were initially studied in *Drosophila* homolog Yki. A landmark report revealed that YAP overexpression in mice leads to dramatic growth in liver size by up to six times and robust development of HCC (Dong et al. 2007). This striking phenotype has attracted substantial scientific interest, highlighting the potential roles of YAP and TAZ in mammalian tissue homeostasis. Specifically, as liver disease progresses, YAP/TAZ levels increase as hepatocytes undergo continuous death and regeneration, which is thought to be related to the disease progression. Since then, YAP/TAZ has been strongly implicated in a wide range of diseases, including metabolic dysfunction-associated steatotic liver disease (MASLD), as discussed in this review.

YAP and TAZ share highly homologous sequences but have some structural variances (Plouffe et al. 2018). YAP is larger than TAZ, and while both contain WW domains that mediate protein–protein interactions, their transcriptional activation domains and binding capacities can differ (Reggiani et al. 2021). Structurally, the TEAD-binding domain of YAP comprises two α-helices connected by a loop containing the PXXΦP motif, whereas TAZ lacks the motif (Chen et al. 2010; Reggiani et al. 2021). They also show some functional differences as well. For example, YAP knockout mice are not viable, showing embryo lethality at embryonic day 8.5 due to defective yolk sac vasculogenesis and embryonic axis development (Morin-Kensicki et al. 2006). In contrast, TAZ knockout mice are viable and fertile albeit they develop renal and lung abnormalities (Hossain et al. 2007; Makita et al. 2008).

In the liver, the Hippo pathway regulates substantial hepatocyte proliferation, differentiation, and survival (Lee et al. 2018). When Hippo signaling is active, its core kinases, namely mammalian sterile 20-like kinase 1 and 2 (MST1/2) and mitogen-activated protein kinase kinase kinase kinase (MAP4Ks), activate large tumor suppressor kinases 1 and 2 (LATS1/2) and induce the phosphorylation of YAP/TAZ at multiple sites (Plouffe et al. 2018). This phosphorylation forces cytoplasmic retention and degradation of YAP/TAZ, thereby preventing its transcriptional activity (Vassilev et al. 2001). Conversely, when Hippo signaling is suppressed, unphosphorylated YAP/TAZ translocates to the nucleus. They interact with the transcription enhancer factor-associated domain (TEAD) family of transcription factors and other transcription partners to regulate the expression of genes crucial for cell proliferation, survival, and metabolic processes (Huh et al. 2019; Jin et al. 2023). Examples of key YAP/TAZ target genes include the cysteine-rich angiogenic inducer 61 (*CYR61*) and connective tissue growth factor (*CTGF*), which are classically known mediators of hepatic inflammation and fibrosis. Therefore, understanding the interplay between YAP/TAZ dysregulation and cellular dysfunction in diverse liver cell types is crucial for the development of novel therapeutic strategies to target complex diseases.

## YAP/TAZ dysregulation in liver diseases

MASLD is defined as a progressive fatty liver disease, developing from simple steatosis to hepatitis, fibrosis, and cirrhosis which eventually causes liver cancer, that meets two of the following metabolic risk factors: obesity, type 2 diabetes, and hyperlipidemia (Eslam et al. 2020). Hepatic steatosis caused by insulin resistance and excess fatty acids can be enhanced by genetic mutations, making it vulnerable to steatohepatitis (Farese et al. 2012). Insulin resistance promotes fat synthesis in the liver and inhibits fat decomposition, causing fatty liver disease and increasing the expression of inflammatory cytokines, causing liver damage and death (Reyes-Gordillo et al. 2017; Chen et al. 2017). In its inflammatory phase called metabolic dysfunction-associated steatohepatitis (MASH), when fat accumulation in hepatocytes becomes severe, structural changes and functional damage to liver tissue occur, leading to fibrosis, and continued fibrosis leads to cirrhosis (Friedman 2024). Additionally, oxidative stress, inflammation, and fibrosis can lead to cirrhosis and hepatocellular carcinoma, which can lead to death from liver disease (Abu Rmilah et al. 2019). MASLD presents a substantial global health challenge, with increasing mortality rates. Two million deaths occur annually from cirrhosis and liver cancer together each year (Asrani et al. 2019). It was not until this year that resmetirom (marketed as Rezdiffra) was approved by the United States Food and Drug Administration drug for the treatment of MASH. It is indicated for use only in relatively mild cases of the F2 stage fibrosis which have not yet progressed to cirrhosis, suggesting that there remains a vast and unmet clinical need (Harrison et al. 2024).

**Fig. 1:**
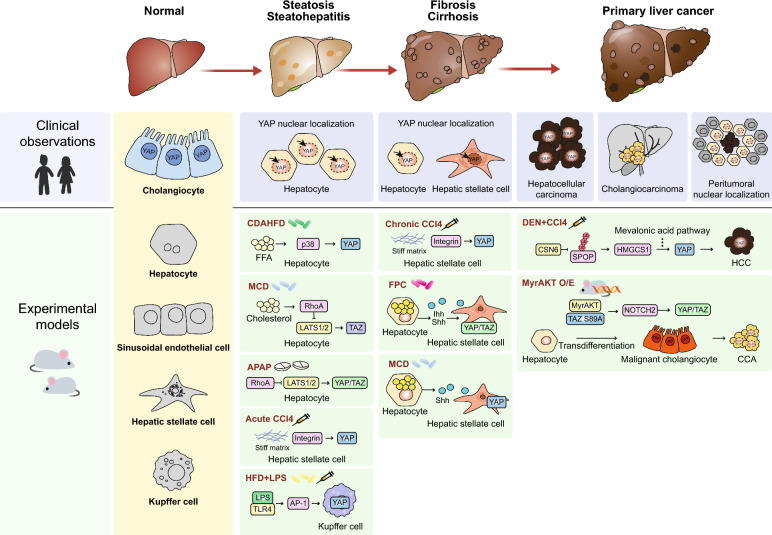
Regulation of YAP/TAZ during MASLD progression in patients and experimental animal models. YAP/TAZ activity during different stages of MASLD. YAP/TAZ expression or activity observed in patients (blue box) and mouse models to mimic human disease (green box) are shown. In physiological conditions, YAP expression is mostly restricted to cholangiocytes in both humans and mice (yellow box). *APAP* acetaminophen; *CCA* cholangiocarcinoma; *CCl*_*4*_ carbon tetrachloride; *CDAHFD* choline-deficient amino acid-defined high-fat diet; *DEN* diethylnitrosamine; *FPC* high-fructose high-palmitate and high-cholesterol diet; *HCC* hepatocellular carcinoma; *HFD* high-fat diet; *LPS* lipopolysaccharide; *MCD* methionine- and choline-deficient diet; *MyrAKT O/E* Hepatocytic overexpression of myristoylated AKT

In the last decade, it has become evident that the aberrant regulation of the Hippo signaling pathway is linked to various aspects of hepatic dysfunction, including changes in the proportion of hepatic cells, altered regeneration capacity, parenchymal death, and inflammation. While initial studies have shown that YAP expression is normally restricted to cholangiocytes in the healthy liver, robust activation of YAP/TAZ was observed in hepatocytes after partial hepatectomy, which is necessary for normal liver regeneration, although not essential (Lu et al. 2018). Subsequent discoveries have demonstrated a substantial increase in YAP/TAZ activity in hepatocytes during acute liver injury, as well as in liver fibrosis (Machado et al. 2015; Wang et al. 2016; Zhang et al. 2016; Kwon et al. 2024). This disease-driven dysregulation of YAP/TAZ appears to be critical for MASLD progression, contributing to hepatocyte dysfunction, inflammation, and fibrosis (Fig. 1).

### Steatosis and steatohepatitis

Steatosis refers to the abnormal accumulation of fat within the liver, typically characterized by fat content exceeding 5% of the liver weight (Nassir et al. 2015). Steatosis commonly arises from metabolic disorders associated with alcohol consumption, obesity, diabetes, and other related conditions. Although the simple accumulation of fat is not regarded as a disease, excess lipids may cause lipotoxicity and induce inflammation, leading to steatohepatitis (Peng et al. 2020). In humans, analysis of liver biopsies from patients with MASH often reveals increased nuclear localization of YAP/TAZ in hepatocytes compared to healthy liver tissues (Mooring et al. 2020; Salloum et al. 2021). Mouse models fed a high-fat diet (HFD) or choline-deficient, amino acid-defined high-fat diet also showed that YAP activation was accompanied by the development of steatosis and steatohepatitis (Salloum et al. 2021). In addition, mice fed a cholesterol-rich, MASH-inducing diet showed increased TAZ expression in hepatocytes (Wang et al. 2020).

Hepatocyte stress is one of the most prominent causes of hepatic YAP/TAZ activation in MASLD. Although direct clinical studies on acute liver injury are less common, elevated YAP expression and nuclear localization have been observed in patients with acute liver failure (Hyun et al. 2019), suggesting that acute hepatocyte damage can trigger substantial YAP/TAZ dysregulation in humans. Studies using animal models have provided strong evidence for YAP/TAZ activation following hepatocyte injury. A recent study has reported aberrant YAP activation in an animal model of acetaminophen-induced liver injury (Kwon et al. 2024). This study revealed that mitochondrial stress activates YAP/TAZ via superoxide-mediated oxidation and activation of Ras homolog family member A (RhoA), a potent activator of YAP/TAZ. Consistent observations have been reported in another mouse model that utilized a single intraperitoneal injection of carbon tetrachloride (CCl_4_), confirming the contribution of oxidative stress to YAP activation (Mannaerts et al. 2015; Verboven et al. 2021). Another study found that pro-inflammatory cytokines such as tumor necrosis factor-alpha (TNFα) can directly modulate hepatocyte YAP activity in a dose-dependent manner, promoting either survival (low TNFα) or death (high TNFα)(Zhao et al. 2020). This highlights a context-dependent regulatory mechanism that adds complexity to the interplay between YAP/TAZ and inflammatory signaling. Notably, high cholesterol levels, which are a characteristic of MASLD, stabilize TAZ. In hepatocytes, cholesterol-induced activation of TAZ is linked to lipid dysregulation and MASLD pathogenesis (Wang et al. 2020). Excessive cholesterol triggers the inositol trisphosphate receptor (IP3R)-calcium-RhoA pathway to activate TAZ.

### Fibrosis and cirrhosis

Liver fibrosis is characterized by the accumulation of extracellular matrix (ECM) proteins. Although it is a regenerative process against hepatocyte loss, excessive ECM production causes liver fibrosis, which can disrupt liver architecture, impair organ function, and alter intrahepatic blood flow. Cirrhosis is an end-stage liver fibrosis characterized by severe architectural distortion and impaired liver function. Accumulating evidence strongly links YAP/TAZ upregulation to the progression of fibrosis and the development of cirrhosis (Machado et al. 2015; Chen et al. 2018; Wang et al. 2020; Zhao et al. 2023). Analysis of liver tissues from patients with fibrosis and cirrhosis repetitively confirmed an increased YAP/TAZ activity compared to healthy livers throughout multiple cohorts. Moreover, the extent of YAP activity is frequently correlated with the severity of fibrosis (Machado et al. 2015; Salloum et al. 2021). Mouse models of liver fibrosis using a methionine and choline-deficient diet, high-fructose, high-palmitate, and high-cholesterol (FPC) diets, or repetitive intraperitoneal injections of CCl_4_ all show increased YAP/TAZ activity accompanied by fibrous scarring and ECM accumulation (Wang et al. 2016; Salloum et al. 2021; Du et al. 2023). In addition to YAP/TAZ activation in hepatocytes upon chronic liver injury, damaged hepatocytes release signals, such as the cytokine Indian Hedgehog (Ihh) and the morphogen Sonic Hedgehog (Shh), and activate YAP/TAZ signaling in hepatic stellate cells (HSCs) as well (Machado et al. 2015; Wang et al. 2016). Increased production of Ihh and Shh has been observed in both human and mouse livers with fibrosis, which was attributed to the enhanced activity of YAP/TAZ in HSCs. Notably, the production of Ihh was shown to be induced by YAP or TAZ in hepatocytes, suggesting its role in intercellular communication.

Increased stiffness of the fibrotic ECM is one of the mechanisms of YAP/TAZ activation owing to their distinct mechanosensitivity (Dupont et al. 2011). A stiff ECM prompts YAP/TAZ nuclear localization and upregulation of the YAP/TAZ target gene, indicating the role of YAP/TAZ as a mechanosensor. Inhibition of YAP reduces the mechanosensitive spontaneous transdifferentiation of HSCs (Mannaerts et al. 2015). YAP/TAZ activation and subsequent HSC transdifferentiation were strongly correlated with alterations in the physical stiffness of the surrounding matrix, as demonstrated using a light-induced secondary crosslinking hydrogel matrix. Excess accumulation of specific ECM proteins such as collagen I and IV is accompanied by liver fibrosis progression. Therefore, changes in ECM composition may also regulate YAP/TAZ through integrin-mediated signaling (Caliari et al. 2016).

### Primary liver cancers

Primary liver cancers refer to cancers that occur in the liver itself, which include HCC and cholangiocarcinoma (CCA). MASLD is a major risk factor, with cirrhosis being the most substantial (Llovet et al. 2021; Ebrahimi et al. 2023). There are several features of MASLD that contribute to the development of cancer. In MASLD, persistent inflammatory signals, endoplasmic reticulum stress, and fatty acid overload induce cell death and DNA damage, which promote the formation of malignant cells. Accumulation of DNA damage from repeated cell death and regeneration can lead to cancerous transformation of liver cells (Matchett et al. 2024). In addition, oxidative stress, or metabolic abnormalities per se can also interfere with physiological regulation of proliferation and tumor suppression, either by alteration of signaling pathways or genetic mutation (Brahma et al. 2021). These changes play an important role in the early stages of primary liver cancer, closely linking MASLD to liver carcinogenesis (Llovet et al. 2021).

Over 60% of human HCC cases exhibit elevated YAP/TAZ expression and nuclear localization, along with increased expression of their target genes. This overexpression correlates with poor prognosis in HCC patients (Zender et al. 2006; Xu et al. 2009; Han et al. 2014; Sohn et al. 2016). Similarly, CCAs display increased YAP/TAZ expression and activity compared with normal bile duct cells (Li et al. 2012; Sugihara et al. 2019). Despite the frequent amplification of YAP/TAZ activity in liver cancers, mutations in the Hippo pathway are rare in HCC and CCA. Therefore, YAP/TAZ signaling may not be the driver of tumor initiation. Nonetheless, unrestrained YAP/TAZ activity is a common feature of many cancers and strongly supports proliferation, invasion, and drug resistance (Guo et al. 2015; Zhou et al. 2019; Cho et al. 2021; Gao et al. 2021; Guegan et al. 2022). Notably, increased nuclear localization of YAP was also observed in normal hepatocytes near liver tumors. Peritumoral activation of YAP/TAZ restrains tumor growth; however, the exact mechanism is unclear (Moya et al. 2019). Similarly, YAP/TAZ has been implicated in the activation of cancer-associated fibroblasts (CAFs) (Calvo et al. 2013). Although this has not been demonstrated in liver cancers, it is probable that hepatic CAFs and HSCs surrounding tumors also have high YAP activity.

Oncogenic signaling pathways found in liver cancers, such as growth factor receptor signaling, Wnt, and Notch, have been reported to promote YAP/TAZ activity to synergize cell proliferation and survival following anticancer treatment (Tschaharganeh et al. 2013; Kim et al. 2017; Moon et al. 2022). For example, synergistic activation of Myc/β-catenin, and YAP/TAZ boosts liver cancer cell proliferation and drug resistance (Bisso et al. 2020). Among the specific upstream inputs in liver cancers, one study suggested that COP9 signalosome subunit 6 (CSN6), a member of the constitutive photomorphogenic 9 (COP9) protein complex implicated in tumorigenesis, drives YAP activation in HCC (Li et al. 2024). In the diethylnitrosamine plus CCl_4_ mouse model, CSN6 antagonizes speckle-type POZ protein (SPOP) and stabilizes hydroxymethylglutaryl-CoA synthase 1 (HMGCS1), resulting in YAP activation through the mevalonate pathway. Hydrodynamic transfection of Myr-Akt with TAZS89A, which increases TAZ activity, accelerates iCCA development via the neurogenic locus notch homolog protein 2 (Notch2) (Cigliano et al. 2022). Pre-existing cirrhosis or advanced fibrosis promotes tumorigenesis in liver cancer (Chakraborty et al. 2017; Roy et al. 2023). In addition to oncogenic signaling pathways, altered matrix stiffness is another major contributor to YAP/TAZ activation, promoting cell proliferation and invasiveness (Yang et al. 2020; Deng et al. 2022). Notably, livers with steatohepatitis that are not considered stiff can promote tumor incidence through altered viscoelasticity, which also causes mechanosensitive YAP/TAZ activation (Fan et al. 2024).

## Cell-type specific roles of YAP/TAZ in liver disease

While YAP/TAZ are primarily expressed in BECs in normal liver, their distribution is observed to spread throughout all major cell types in the liver during the progression of MASLD. As liver disease progression is orchestrated by different cells in the liver, dissecting the cell type-specific role of YAP/TAZ is crucial to develop effective treatment and management strategies for MASLD.

### Hepatocytes

**Fig. 2:**
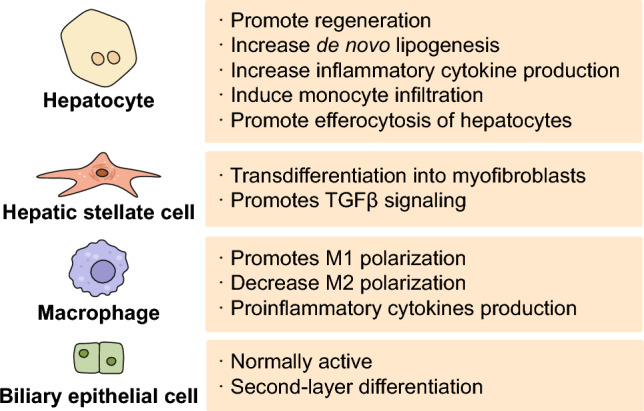
Cell type-specific functions promoted by YAP/TAZ. Upon activation, YAP/TAZ exerts distinct cellular effects in both parenchymal and non-parenchymal cells that comprise the liver. The boxes show the YAP/TAZ-dependent functions that have been identified in the indicated cell type to date

Hepatocytes account for 80% of the liver mass and are involved in the major functions of the liver, such as protein synthesis, metabolism of stored carbohydrates, synthesis of cholesterol and bile juice, and detoxification and excretion of substances (Fabris et al. 2019). YAP/TAZ activity affects hepatocyte proliferation, lipid metabolism, inflammation, and fibrosis, demonstrating its multifaceted effect on liver pathophysiology (Fig. 2). The contribution of YAP to liver homeostasis is represented by the significant hepatomegaly observed in mice with hepatocyte-specific overexpression of active mutant YAP (Dong et al. 2007). After massive tissue loss, YAP/TAZ is activated in a controlled and transient manner in the remaining hepatocytes (Lu et al. 2018). This accelerates hepatocyte proliferation, as demonstrated in mouse models, where partial hepatectomy leads to the rapid nuclear localization of YAP and the subsequent induction of target genes involved in cell proliferation (Wu et al. 2013). Conversely, the deletion of YAP/TAZ in hepatocytes leads to an impaired rate of tissue regeneration. The pharmacological inhibition or genetic deletion of MST1/2 therefore enhances liver regeneration, highlighting the pharmacological value of modulating the Hippo pathway (Lu et al. 2018). Interestingly, however, the mice lacking YAP/TAZ are still able to fully repopulate the liver, implying the existence of additional compensatory machineries (Verboven et al. 2021). Although promoting hepatocyte proliferation is generally beneficial for repopulating tissues, the precise consequences of YAP/TAZ activity depend on the overall health of the liver microenvironment. For example, sustained or excessive YAP overexpression in an injured liver may paradoxically lead to the elimination of proliferating hepatocytes as part of the injury response (Miyamura et al. 2017).

YAP/TAZ dysregulation in hepatocytes plays a multifaceted role in driving steatosis and inflammation, thereby contributing to the progression of MASLD. A key mechanism contributing to steatosis involves the direct regulation of sterol regulatory element-binding proteins (SREBPs) in hepatocytes, leading to increased lipid synthesis and accumulation (Wang et al. 2016). The components of the upstream Hippo pathway also affect this process. For example, inhibition of MST1/2 or LATS1/2 enhances SREBP activity and promotes fatty liver disease (Aylon et al. 2016; Lu et al. 2018). Furthermore, MST1 influences lipid metabolism by promoting the expression of Sirt1, a negative regulator of lipid synthesis (Zhou et al. 2019). Although specific investigations into the link between YAP/TAZ and inflammation in MASLD are warranted, insights from general liver injury models suggest that YAP dysregulation in hepatocytes promotes the production of pro-inflammatory cytokines that exacerbate the disease (Mooring et al. 2020). For example, acute hepatocyte injury caused by intraperitoneal injection of CCl_4_ induces CYR61 expression through YAP/TAZ to promote pro-inflammatory monocyte infiltration associated with steatohepatitis (Noguchi et al. 2018).

The dysregulation of YAP/TAZ in hepatocytes also contributes to the development of fibrosis. Hepatocyte-specific YAP-knockout mice are resistant to the development of liver fibrosis induced by chronic CCl_4_ administration (Mooring et al. 2020). In the same study, deletion of both YAP and TAZ did not further inhibit the development of fibrosis, indicating that YAP, but not TAZ, is the primary contributor. Another study has reported that elevated TAZ activity induces the production of Ihh, a potent activator of HSCs, and exaggerates diet-induced fibrosis driven by an FPC diet (Wang et al. 2016). A separate study by the same group demonstrated that hepatocyte-specific N-acetylgalactosamine-conjugated siRNA targeting TAZ was sufficient to ameliorate diet-induced liver fibrosis in mice (Wang et al. 2019). Further studies are warranted to determine how YAP and TAZ differentially contribute to MASLD in humans.

### Hepatic stellate cells

HSCs reside in the space of Disse, a region between the liver sinusoidal endothelial cells and hepatocytes (Wake 1971). In the normal liver, HSCs maintain a quiescent state, retaining retinyl esters, representing up to 10% of all liver-resident cells. Lineage-tracing studies have identified HSCs as the primary cause of liver fibrosis, except for cholestatic injury (Mederacke et al. 2013; Iwaisako et al. 2014). Upon liver injury, these cells transdifferentiate into myofibroblast-like cells. Activated HSCs secrete ECM proteins and pro-inflammatory cytokines with enhanced proliferative, migratory, and contractile phenotypes (Tsuchida and Friedman 2017). Accumulating evidence implicates YAP/TAZ as crucial modulators of HSC activation. Transcriptomic analysis has revealed that the upregulation of YAP target genes is an early hallmark of activation, occurring both in cell culture models and in vivo during liver injury (Mannaerts et al. 2015; De Smet et al. 2021). During liver injury, multiple stimuli converge to promote nuclear translocation and transcriptional activity of YAP/TAZ in HSCs. These factors regulate the expression of genes crucial for HSC proliferation (e.g., CTGF and CYR61) and survival (e.g., BIRC5) along with the production of ECM components, such as collagen I and alpha-smooth muscle actin (α-SMA). Mechanistically, stiffening of the ECM, which occurs in fibrotic livers, efficiently promotes YAP/TAZ nuclear localization in HSCs (Caliari et al. 2016). YAP/TAZ also forms an autocrine feed-forward loop in HSCs. For instance, YAP/TAZ can promote transforming growth factor-beta (TGF-β) signaling, which further reinforces YAP/TAZ activity, as demonstrated by enhanced SMAD2/3 phosphorylation upon YAP/TAZ overexpression (Haak et al. 2019). Most importantly, the necessity of YAP/TAZ in the pro-fibrotic function of HSCs has been demonstrated in studies where pharmacological inhibition with verteporfin attenuated HSC activation and collagen production in vitro and reduced liver fibrosis in mouse models (Mannaerts et al. 2015; Martin et al. 2016; Du et al. 2018; Liu et al. 2019).

YAP/TAZ also plays a crucial role in regulating the fate of HSCs. Specifically, YAP enhances the sensitivity to ferroptosis while conferring resistance to senescence. Silencing of YAP and TAZ renders HSCs susceptible to ferroptosis and promotes senescence. Notably, HSC-specific YAP knockout in the mouse leads to an increase in senescent HSCs and offers protection against liver fibrosis, as evidenced by reduced expression of αSMA and decreased collagen accumulation (Du et al. 2023). Collectively, these findings highlight the multifaceted role of YAP/TAZ in HSCs and their potential as therapeutic targets in liver fibrosis.

### Hepatic macrophages

Hepatic macrophages comprise resident macrophages known as Kupffer cells (KCs), and macrophages derived from circulating monocytes (Wen et al. 2021). Hepatic macrophages play a crucial role in maintaining liver homeostasis and repair. Although there is little evidence regarding the role of YAP/TAZ in hepatic macrophages, several reports have suggested their regulatory potential (Thomann et al. 2021). In a MASH mouse model, YAP expression in KCs promoted M1 polarization and increased the production of inflammatory cytokines (Song et al. 2020). In addition, the deletion of YAP in KC, HFD, and lipopolysaccharide (LPS) treatment resulted in the inhibition of M1 polarization and an increase in the M2 proportion in the HFD plus LPS model of NASH in mice. However, its role in KCs seems to be restricted to the regulation of the inflammatory response and not to hepatic metabolism, since it does not alter hepatic lipid accumulation (Song et al. 2020). Another study showed that increased YAP expression in KC promotes the secretion of inflammatory cytokines, leading to liver damage (Liu et al. 2024). This demonstrated that YAP plays an important role in the regulation of liver injury and inflammation through interactions between hepatic cells. Furthermore, YAP/TAZ interplay with other signaling pathways, notably Notch1 and TGF-β, in regulating macrophage activation and function in the liver milieu (Feng et al. 2018; Yang et al. 2023). Such crosstalk may contribute to fine-tuning the balance between the pro- and anti-inflammatory responses.

### Biliary epithelial cells

BECs, also known as cholangiocytes, line the bile ducts in the liver and play a critical role in liver function and pathology (O'Hara et al. 2013). Physiologically, BECs regulate bile acid composition and flow (Hrncir and Gracz 2023). Disruption in bile acid homeostasis upon malfunction of BECs can contribute to liver injury and inflammation, which are central to the pathogenesis of MASLD (Chiang and Ferrell 2018). Excess bile acids exert direct cytotoxic effects that can exacerbate hepatocyte injury in steatohepatitis and influence lipid metabolism. This inflammatory response can further drive hepatocyte damage and fibrosis, which are hallmarks of steatohepatitis. Moreover, in conditions where chronic inflammation is present, activated BECs secrete profibrogenic cytokines and growth factors that promote the activation of hepatic stellate cells, ultimately leading to fibrosis (Poulsen et al. 2022).

Enforced expression of S127-YAP or deletion of NF2 causes hepatocyte dedifferentiation into Sox9- or CK19-expressing BEC-like cells. This process is dependent on Notch signaling. Notably, dedifferentiated cells were able to re-differentiate into hepatocytes when the expression of S127A-YAP was reversed, indicating a role for YAP in hepatic cell plasticity (Yimlamai et al. 2014). BECs are epithelial cells that surround the biliary tubules and transport bile from the liver to the small intestine (Tabibian et al. 2013). Recent studies have demonstrated the significance of YAP/TAZ in the regulation of BECs proliferation and maintenance. For instance, deletion of YAP in adult liver cells results in bile duct paucity and delayed regeneration or hepatocyte necrosis (Airik et al. 2020; Verboven et al. 2021). The role of YAP in early biliary development is important because YAP is required for the differentiation of the second layer of BECs (Molina et al. 2021, 2022). Notably, TAZ overexpression promotes the conversion of lipid-rich hepatocytes into fully malignant BECs in combination with AKT (Cigliano et al. 2022). YAP has been reported to be a key feature of CCA, and YAP activity is associated with a poor overall survival rate (Pei et al. 2015; Sugimachi et al. 2017; Toth et al. 2021).

## Drug candidates targeting YAP/TAZ in the liver

The discovery and development of small-molecule inhibitors and activators targeting the YAP/TAZ signaling pathway have emerged as a promising strategy in cancer research. A previous study reported the discovery of a small molecule inhibitor called verteporfin, the most widely used to date, which effectively suppressed YAP/TAZ signaling and was confirmed to inhibit YAP-induced liver overgrowth (Liu-Chittenden et al. 2012). Pharmacological inhibition of YAP by verteporfin enhances the sensitivity of cancer cells to sorafenib and overcomes tumorigenesis (Sun et al. 2021). The combination of sorafenib and verteporfin substantially inhibits HCC growth. CA3 is another small molecule that inhibits YAP and has been proposed as an anticancer agent against HCC (Han et al. 2022). Enhanced expression of cyclin-dependent kinase 6 (CDK6) has been proposed as one of the main causes of lenvatinib resistance in HCC (Leung et al. 2023; Jing et al. 2023). A recent study showed that CA3 effectively inhibits CDK6 expression, demonstrating the potential of YAP/TAZ inhibitors in combination therapy for HCC (Leung et al. 2023). As a common mechanism, these inhibitors prevent the interaction between YAP/TAZ and TEAD, thereby inhibiting YAP/TAZ-mediated transcription (Table 1). In addition, GalNAc-siTAZ can be another viable therapeutic given the current acceptance of nucleic acid medicines. Conjugation of a GalNAc ligand targeting the asialoglycoprotein receptor with chemically modified siRNA resulted in robust and durable RNAi-mediated TAZ silencing specifically in hepatocytes (Debacker et al. 2020). Systemic injection of GalNAc-siTAZ reduced liver inflammation, hepatocyte damage, liver fibrosis, and profibrotic mediator expression compared to the GalNAc-control group (Wang et al. 2023).

**Table 1:** Inhibitors of YAP/TAZ that have been shown to work in the liver

Generic name	Molecular formula	CAS no	Molecular structure	Clinical trials	References
***Small molecules***					
Verteporfin	C_41_H_42_N_4_O_8_	129497-78-5		NCT00403442 NCT01968486	Sun et al. (2021)
CA3	C_23_H_27_N_3_O_5_S_2_	300802-28-2		–	Han et al. (2022)
***Antisense oligonucleotide***					
GalNAc-siTAZ*	–	–		–	Wang et al. (2019) Wang et al. (2023)

Therapeutic modulation of YAP/TAZ is expected to require a delicate balance. The challenge lies in inhibiting the pathological amplification of YAP/TAZ signaling in liver cells without impairing the regenerative capacity of the liver or stimulating regeneration without promoting tumorigenesis. The development of dual modulators that can inhibit or activate YAP/TAZ signaling in a context-dependent manner may provide more viable therapeutic options. While both inhibitors and activators have the potential to contribute substantially to the management of liver tumors and other complications, such as MASLD, the discovery and optimization of such modulators of YAP/TAZ signaling represents a promising frontier in the treatment of liver disease.

## Conclusion

The intricate interplay between YAP/TAZ and liver dysfunction emphasizes their significance in MASLD. Based on their regulation of lipid metabolism, inflammation, and fibrosis, and their impact on different types of cells in the liver, YAP/TAZ have emerged as central players governing diverse aspects of liver homeostasis and disease progression. In MASLD, aberrant activation of YAP/TAZ is intricately linked to hepatocyte stress, steatosis, inflammation, and fibrosis, representing a critical nexus throughout disease progression. Furthermore, their dysregulation contributes to the development and progression of MASLD and primary liver cancer. Understanding the cell type-specific roles of YAP and TAZ in different liver cell populations, including hepatocytes, HSCs, hepatic macrophages, and BECs, is crucial to deciphering their diverse functions in liver physiology and pathology.

It is important to note that this review should be interpreted with caution. In particular, the experimental models using mice may not fully mimic the human condition of MASLD, potentially limiting the applicability of the findings to clinical settings. The complex interplay of (epi)genetic and environmental factors in clinical settings may introduce unaccounted variables that could influence the outcomes that cannot be explained by the currently used experimental models. In addition, given the incomplete understanding of the mechanism by which YAP/TAZ are redistributed and activated in different hepatic cells during MASLD, it is inadvisable to rely on a single general mechanism for YAP/TAZ regulation. In a similar context, considering the importance of the interplay between YAP/TAZ and MASLD progression, identification of their upstream input(s) will be the next aim for developing therapeutic modulators. For instance, studies on the signaling pathways linked to YAP/TAZ activity have identified the mechanical properties of the extracellular matrix as an emerging target. Consequently, the regulation of the mechanosensitive activation of YAP/TAZ may be an important research topic for understanding the differences between healthy and diseased tissues in MASLD.

In summary, the elucidation of YAP/TAZ-mediated molecular mechanisms and their intricate involvement in liver pathophysiology not only enhances our understanding of liver diseases but also paves the way for the development of innovative therapeutic strategies aimed at addressing unmet clinical needs in the field of hepatology.
